# Factors associated with permanent hypothyroidism in infants with congenital hypothyroidism

**DOI:** 10.1186/s12887-019-1833-8

**Published:** 2019-11-22

**Authors:** Eun Sil Park, Ju Young Yoon

**Affiliations:** 1 Department of Pediatrics, Gyeongsang National University College of Medicine, Jinju, South Korea; 2 Gyeongsang Institute of Health Science, Gyeongsang National University College of Medicine, Jinju, South Korea; 3 Department of Pediatrics, Pusan National University Children’s Hospital, Yangsan, South Korea; 4 Department of Pediatrics, Gyeongsang National University Changwon Hospital, Changwon, South Korea

**Keywords:** Congenital hypothyroidism, Levothyroxine, Risk factor, Prognosis

## Abstract

**Background:**

Congenital hypothyroidism (CH) is one of the most common endocrine diseases in childhood. A significant proportion of CH cases are transient, but the risk factors for permanent CH (PCH) are not yet well established. The current guidelines suggest using levothyroxine until the age of 3 years, but some studies suggest the possibility of earlier discontinuation. However, few, if any, studies have followed up on the results of early discontinuation. This study aimed to identify predictive factors of transient CH among infants with CH. We also investigated the results in patients who underwent a trial of early discontinuation.

**Methods:**

We gathered data regarding infants diagnosed with CH between July 2005 and July 2015 by retrospective chart review. Those with aplastic, hypoplastic or ectopic glands on thyroid ultrasonography or scan were excluded. Among them, early discontinuation subgroup was defined as those who discontinued levothyroxine before 30 months of age.

**Results:**

From the 80 infants (40 males, 40 females) enrolled in this study, 51 were preterm. Nine (11.3%) were diagnosed with PCH. Compared with transient cases, those with PCH were on higher levothyroxine dose at discontinuation (4.3 vs 2.9 μg/kg, *P* < 0.001). There was no difference in the proportion of permanent cases between preterm and full-term groups. In preterm group,infants with PCH required higher levothyroxine dose at discontinuation than those with transient CH (3.8 vs 2.5 μg/kg, *P* = 0.018). Levothyroxine discontinuation at a dose of 2.86 μg/kg could suggest PCH (sensitivity, 88.9%; specificity, 71.0%). Among the 9 patients who underwent a trial of early discontinuation, 8 successfully discontinued levothyroxine.

**Conclusion:**

The majority of CH patients discontinued levothyroxine successfully, including those who underwent a trial of early discontinuation. Higher levothyroxine dose at the time of discontinuation was found to be a predictive factor for PCH.

## Background

Congenital hypothyroidism (CH) is one of the most common endocrine diseases among children, and can cause intellectual impairment [1]. In many cases, CH results from transient abnormalities in the thyroid function rather than permanent dysfunction [2], but current guidelines recommend that levothyroxine treatment be maintained until at least 36 months of age for all infants diagnosed with CH [3]. For parents and infants, taking medication every day for 3 years and undergoing routine blood sampling for follow-up thyroid function tests (TFTs) are difficult tasks. In the United States, more than one-third of children undergoing treatment for CH discontinue treatment within 36 months, some without any medical advice [2]. In addition, the recent evidence suggests that exposure to excess thyroid hormone may be as harmful as hypothyroidism to long-term cognitive development [4, 5]. Therefore, reasonable, individualized, and easy-to-follow guidelines for early discontinuation are needed. Thus, it would be possible to try early discontinuation, especially when there is a high possibility that the patient is experiencing transient CH (TCH).

Several studies have investigated the predictors of TCH. Hypothyroidism is more common among preterm infants than among full-term infants, but a higher proportion of preterm infants with CH may have TCH than full-term infants [6]. The levothyroxine dose at discontinuation trial was also identified as a predictor for permanent CH (PCH) [7, 8]. Several studies proposed possible early discontinuation in some cases, such as those with low levothyroxine dose [9] or preterm infants [10]. However, these were retrospective studies involving a relatively small number of infants, so there is no consensus on the predictors of transient hypothyroidism.

In this study, we investigated the differences between transient and permanent CH groups. We also examined the clinical characteristics and results of infants who underwent a trial of early discontinuation; we tried to identify the predictors of TCH to identify which patients are good candidates to try early discontinuation.

## Methods

### Subjects

The subjects were Korean infants with CH born in our hospital or referred to our hospital. Inclusion criterias were infants who were diagnosed as CH between July 2005 and July 2015, started levothyroxine before 3 months of age, and underwent TFTs for more than 6 months after discontinuation of the treatment. Those with aplastic, hypoplastic or ectopic glands on thyroid ultrasonography or scan were excluded. Among those who were enrolled, SONO and thyroid scan was done in 40 and 39 patients, respectively.

We collected data regarding the patients’ basic demographics, including gestational age, birth weight, sex, age, and weight at each visit. We also collected data regarding the results of neonatal screening and TFTs, and levothyroxine dose.

Neonatal screening tests (NSTs) were performed 2–4 days after birth in full-term infants and within 7 days in preterm infants as per the protocol [11]. NSTs were repeated for all preterm infants or term infants with NST thyroid stimulating hormone (TSH) levels above the cutoff value. A TFT was performed if in the repeat NST, the level of the TSH was abnormal. All TFTs among preterm infants were performed at least 3 times at the ages of 7 days, 2–4 weeks, and prior to discharge from the neonatal intensive care unit. Follow-up tests in outpatient pediatric endocrinology clinics were performed as needed. TSH and free T4 (fT4) levels were measured in peripheral venous blood samples using Electrochemiluninescenceimmunoassay(ECLA) (Roche Diagnostics Ltd.,Swiss) as per manufacturer’s protocol. Hypothyroidism was diagnosed if the fT4 level was below 0.9 ng/dl or if the TSH level was above the cutoff value (> 20 μU/ml at any time or > 10.0 μU/ml after 4 weeks of age). Delayed TSH elevation was defined when initial NST TSH level was below 20 μU/ml, but serum TSH level was elevated (> 20 μU/ml).

Levothyroxine treatment was initiated after the diagnosis of hypothyroidism, at an initial dosage of 10–15 μg/kg/day. The levothyroxine dosage was adjusted according to the follow-up TFT results. Trial of discontinuation was performed between the ages of 2.5 and 3 years, but some parents stopped treatment without being advised to do so. Follow-up TFTs were performed at 1, 6, and 12 months after discontinuation of levothyroxine. PCH was diagnosed if the fT4 level was below 0.9 ng/dl or if the TSH level was above > 10.0 μU/ml, and levothyroxine was restarted Normal TFT results for up to 12 months after discontinuation of levothyroxine confirmed the diagnosis of TCH.

### Statistical analysis

The statistical analyses were performed using SPSS Statistics version 21.0 (IBM Corp., Armonk, NY, USA). The results were expressed as mean and median values, and variability was indicated by the standard deviation and/or range. Continuous data were analyzed using the student’s *t*-test or the Mann-Whitney *U* test, and categorical variables were analyzed using the *χ*^2^ test or Fisher’s exact test.

We investigated multicollinearity using the variance inflation factor. The variance inflation factor was 1.299, which implied a lack of multicollinearity, so these data were adequate for logistic regression analysis. Thus, logistic regression was performed to identify the predictors of PCH.

To evaluate the optimum cutoff levels of predictors, we performed receiver operating characteristic (ROC) analyses with PCH as the dependent variable. Results with *P* < 0.05 were considered significant.

## Results

### Patients

A total of 80 infants were enrolled in this study (40 males and 40 females). The mean gestational age was 33.6 ± 4.6 weeks, and the mean birth weight was 2.1 kg. Levothyroxine discontinuation failed in 9 infants (11.3%) and they were diagnosed with PCH (PCH group), while the rest (71, 88.8%) successfully discontinued levothyroxine (TCH group). Nine (11.3%) patients tried levothyroxine discontinuation before 30 months of age (the early discontinuation group), and all the others between 30 and 36 months (the on-time discontinuation group). The clinical characteristics of all participants and subgroups are described in Table 1.

**Table 1:** Demographic and auxologic characteristics of participants^a^

Characteristic	All patients (*n* = 80)	Off trial success (*n* = 71)	Off trial failure (*n* = 9)	*P*	Early off trial (n = 9)	On-time off trial (n = 71)	*P*
Male, n (%)	40 (50.0)	35 (49.3)	5 (55.6)	1	5 (55.6)	35 (49.3)	1
GA (weeks)	33.6 ± 4.6	33.6 ± 4.5	34.0 ± 5.8	0.835	33.6 ± 4.5	33.7 ± 4.7	0.95
Age (treatment initiation, weeks)	3.4 ± 3.1	3.2 ± 2.3	5.5 ± 6.8	0.349	3.0 ± 2.5	3.5 ± 3.2	0.687
Age (discontinuation trial, months)	34.5 ± 4.6	34.6 ± 4.4	33.9 ± 5.9	0.67	23.7 ± 3.9	35.9 ± 2.2	< 0.001
Wt (at birth, kg)	2.1 ± 0.9	2.0 ± 0.9	2.3 ± 1.1	0.469	2.1 ± 0.8	2.1 ± 0.9	0.956
Wt (at treatment initiation)	2.5 ± 1.0	2.4 ± 1.0	3.1 ± 1.3	0.094	2.3 ± 0.9	2.5 ± 1.0	0.518
Wt (at discontinuation)	12.8 ± 1.8	12.8 ± 1.8	13.4 ± 1.8	0.287	11.7 ± 1.6	13.0 ± 1.8	0.047

Abbreviations: Wt, weight; GA, gestational age

^a^Quantitative data are expressed as the mean ± SD (standard deviation), and qualitative data are expressed as frequency (%)

^*^P < 0.05

### Off trial results

A higher proportion of infants in the PCH group had TSH level > 20 μU/ml on NST compared to the TCH group (62.5% vs 7.0%, *P* = 0.001). Infants in the PCH group also had higher levothyroxine dose per weight at 1 year, 2 years, and off trial than the TCH group (4.3 vs 2.5, 4.9 vs 3.5, and 4.3 vs 2.9 μg/kg, respectively). Three children increased the dose during 2–3 years and all of them were PCH group (data not shown).

There were no differences in fT4 and TSH levels between the two groups, neither in starting nor discontinuing medication Among 20 patients who showed delayed TSH elevation, all except one succeeded to discontinue levothyroxine (Table 2).

**Table 2:** Laboratory findings and levothyroxine dose^a^

Characteristic	All patients (n = 80)	Off trial failure (n = 9)	Off trial success (n = 71)	*P*	Early off trial (n = 9)	On-time off trial (n = 71)	*P*
NST							
TSH (μU/ml) (median,range)	5.2 (0.1–356)	31.3 (0.8–260)	4.8 (0.1–356)	0.141	5.3 (0.1–356)	4.8 (0.5–11.9)	0.525
T4 (μg/dl)	6.8 ± 3.2	7.6 ± 4.1	6.7 ± 3.2	0.557	7.3 ± 1.9	6.7 ± 3.4	0.674
TSH > 20 μU/ml (n,%)	9 (13.8)	5 (62.5)	4 (7.0)	0.001*	0	9 (15.3)	0.584
T4 < 5 μg/dl (n,%)	12 (21.8)	1 (20.0)	11 (22.0)	1	0	12 (24.0)	0.574
Initial TSH (μU/ml) (median,range)	17.4 (0.8–100.0)	43.2 (1.0–100.0)	17.4 (0.8–100.0)	0.075	17.6 (0.8–199.9)	7.2 (5.0–42.0)	0.009*
Initial fT4 (ng/dl)	1.2 ± 0.4	1.0 ± 0.4	1.2 ± 0.4	0.437	1.3 ± 0.2	1.1 ± 0.4	0.162
TSH > 20 μU/ml (n,%)	39 (49.4)	5 (55.6)	34 (48.6)	0.737	5 (55.6)	34 (48.6)	0.737
fT4 < 0.9 ng/dl (n,%)	22 (30.1)	2 (40.0)	20 (29.4)	0.634	0 (0)	22 (34.4)	0.049^*^
TSH at off trial (μU/ml)	3.4 ± 3.0	5.7 ± 3.7	3.1 ± 3.7	0.295	3.8 ± 2.3	3.4 ± 3.1	0.682
Delayed TSH elevation (n,%)	20(25.0)	1(11.1)	19(26.8)	0.437	2(22.2)	18(25.4)	1.000
fT4 at off trial (ng/dl)	1.5 ± 0.2	1.5 ± 0.2	1.5 ± 0.2	0.796	1.4 ± 0.2	1.5 ± 0.2	0.138
Initial T4 dose (μg/kg/day)	11.2 ± 2.5	10.7 ± 2.5	11.4 ± 2.5	0.442	12.1 ± 2.0	11.2 ± 2.5	0.306
T4 dose (1 year) (μg/kg/day)	3.7 ± 1.4	4.3 ± 1.4	2.5 ± 1.4	< 0.001	3.3 ± 1.7	3.7 ± 1.3	0.419
T4 dose (2 years) (μg/kg/day)	3.1 ± 1.2	4.9 ± 1.2	3.5 ± 1.2	0.002	2.4 ± 0.3	3.2 ± 1.2	0.234
T4 dose at off trial (μg/kg/day)	2.8 ± 1.2	4.3 ± 1.2	2.9 ± 1.2	0.001	2.5 ± 0.7	2.8 ± 1.2	0.44
Off trial failure (n,%)	9 (11.3)	9 (100%)	0 (0%)	–	1(11.1)	8(11.3)	1.000

Abbreviations: Wt, weight; GA, gestational age; TSH, thyroid stimulating hormone; T4, thyroxine; fT4, free thyroxine; NST, neonatal screening test

^a^Quantitative data are expressed as the mean ± SD (standard deviation) or median (range), and qualitative data are expressed as frequency (%)

^*^P < 0.05

The early discontinuation group had lower initial TSH levels than the on-time discontinuation group (17.5 vs 33.5 μU/ml, *P* 0.009) and none of the patients in the early discontinuation group had fT4 levels below the 0.9 ng/dl (Table 2).

### Predictive factors for treatment failure

We performed binary logistic regression analysis with abnormal TSH level on NST and levothyroxine dose at discontinuation as independent variables, and discontinuation failure as the dependent variable. The result showed that the levothyroxine dose at discontinuation was a significant predictor of discontinuation failure (odds ratio 3.443, *P* 0.009). The power of explanation of the model was 37.4% (Table 3).

**Table 3:** Results of binary logistic regression analysis of factors associated with transient congenital hypothyroidism (n = 80, *R*^2^ = 0.258)

Variable	*β*	Standard error	Wald statistic	*P*	Odds ratio
Constant	−5.884	1.769	11.058	< 0.001	0.003
T4 dose at off trial	1.028	0.483	4.522	0.033	2.795
NST TSH > 20 μU/ml	1.811	1.077	2.830	0.093	6.119

Abbreviations: NST, neonatal screening test; TSH, thyroid stimulating hormone; T4, thyroxine

**Fig. 1:**
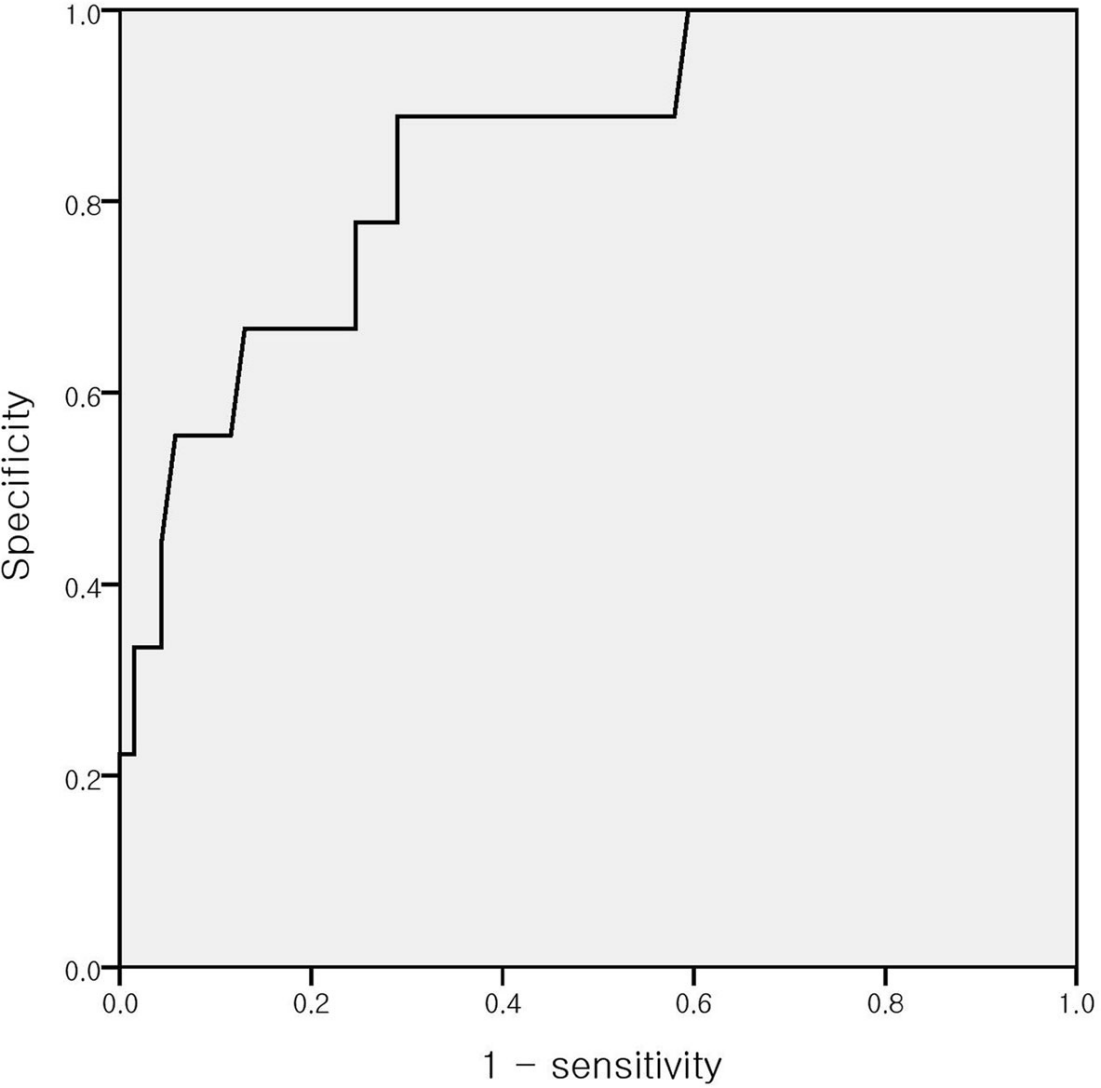
Receiver operating characteristic curve of various thresholds of levothyroxine for predicting transient congenital hypothyroidism. A levothyroxine dose of 2.86 μg/kg at the off trial may lead to discontinuation failure with a sensitivity of 88.9% and specificity of 71.0%, and an area under the ROC curve of 0.8

We plotted a ROC curve to identify the cutoff dose of levothyroxine at discontinuation suggestive of off trial failure. A levothyroxine dose of 2.86 μg/kg could suggest discontinuation failure with a sensitivity of 88.9% and specificity of 71.0%, and an area under the ROC curve of 0.849 (Fig. 1).

### Preterm infants

Fifty-one (63.8%) of all participants were born preterm. The demographic and clinical characteristics of the preterm and term groups are described in Additional file 1: Table S1. Term infants had higher initial TSH levels than the preterm group (44.6 vs 24.5 μU/ml, *P* = 0.016), but there was no difference in discontinuation failure rate (17.2 vs 7.8%, *P* = 0.273) (Additional file 1: Table S2).

We investigated the difference between PCH and TCH infants in preterm infants. The TCH group had lower levothyroxine dose at discontinuation than the PCH group (2.5 vs 3.8 μg/kg, *P* = 0.018) (Additional file 1: Table S3).

## Discussion

In this study, infants in the discontinuation success group received lower levothyroxine doses during the treatment period than subjects in the discontinuation failure group. Furthermore, the levothyroxine dose at discontinuation was significantly associated with discontinuation failure. A dose of 2.86 μg/kg at discontinuation was the optimal cutoff value that could predict discontinuation failure.

In a previous study conducted by Messina et al. [12] the prevalence of TCH was 36.5%; however, subjects with ectopic thyroid gland were also included in the study. Ghasemi et al. [13] reported that 79.4% of patients with primary CH had TCH, and the prevalence of TCH was 1 in 294 live births. In a study conducted by Eugster et al. [14], among 33 children with primary CH (including 9 with absent or ectopic thyroid), 12 (36%) had TCH. In previous Korean studies, the proportion of TCH among CH patients ranged from 39.4 to 65.0% [6, 7, 9, 15]. In our study, 89.7% of patients with CH were diagnosed with TCH. This high proportion is partially explained by the fact that our study excluded those with ectopic thyroid or thyroid aplasia. Another reason is that our study included a high proportion (63.8%) of preterm infants, among whom transient hypothyroidism is reportedly more common than among full-term infants [16, 17].

The levothyroxine dose required to maintain normal thyroid function is known to be lower in the TCH group than in the PCH group [12, 18, 19], and several studies suggested the use of levothyroxine dose during treatment or at discontinuation as a predictor of PCH. Rabbiosi et al. reported that daily T4 requirement above 2 μg/kg was a predictor of PCH [8]. Similarly, Lee et al. reported that T4 requirement lower than 2.76 μg/kg/day could predict TCH [7]. In our study, the levothyroxine dose at the third year of treatment was a positive predictor of TCH diagnosis, with a cutoff value of 2.86 μg/kg, which was similar to that reported in previous studies.

It is controversial whether the laboratory finding can predict TCH. Some previous studies suggested that children with TCH had significantly lower initial TSH levels compared to those with PCH [7, 10, 18]. However, other studies have reported that the initial fT4 and TSH levels were not different between TCH and PCH cases [6, 8, 12]. In our study, abnormal NST TSH levels (> 20 μU/ml) were more common in the PCH group than in the TCH group, but initial serum TSH levels showed no difference.

Hypothyroidism is more common among preterm infants than among full-term infants [16, 17]. However, preterm infants with high TSH levels may have TCH rather than PCH, and early reevaluation can be particularly necessary for these patients [17]. In our study, there was no difference in the proportion of TCH patients between the term and preterm groups. It is known that delayed TSH elevation is common in preterm infants, and these patients generally have transient CH [20]. In our study, 31.4% of preterm babies showed delayed TSH elevation, and only one of preterms with delayed TSH elevation failed to discontinue levothyroxine. Few, if any, previous studies have followed up the results of early discontinuation trial. In a study conducted by Lim et al., 39 infants with very low birth weight discontinued L-T4 therapy at around 2 years of age, all of whom retained normal thyroid function without medication [10]. In our study, among 9 patients who tried to discontinue levothyroxine early (before 30 months of age), all except one successfully discontinued treatment. Our study showed that in CH infants with eutopic thyroids and only mildly elevated TSH on NST, the majority can successfully discontinue L-T4 by 3 years of age. Our study also suggests that early discontinuation could be tried in selected patients.

One of the strengths of our study is that it involved a relatively large number of infants, including both full-term and preterm infants. Another strength is that this was a single center study, including only those with eutopic thyroid glands, to minimize differences between the groups. And we compared the characteristics of PCH and TCH group in preterm infants, which has not been investigated.Also, we described the results of early discontinuation trial, though the number of patients was small.

The limitation of our study is that it was retrospective. It is possible that children in the early discontinuation group tried early discontinuation because their thyroid function was controlled successfully. However, there were no significant differences in levothyroxine dose or laboratory findings during treatment between the two groups. The TCH rate might have been underestimated because we included only those who took levothyroxine until 30 months of age. And the number of early discontinuation group is small, so we couldn’t draw success rate of early discontinuation or postulate predictive factor of early discontinuation success. Also, long-term follow-up of cognitive function and growth is necessary to compare long-term consequences between the groups. Nevertheless, our study provide useful data that support a trial of early discontinuation with low levothyroxine requirement, in both preterm and term infants.

## Conclusions

We found that the majority of infants with CH, including those who underwent early trial of discontinuation, successfully discontinued levothyroxine. The levothyroxine dose at the time of discontinuation seems to be associated with permanent hypothyroidism. Early discontinuation with careful monitoring of thyroid function would be an option for those receiving low levothyroxine dose.

## Supplementary information

**Additional file 1: Table S1.** Demographic and auxologic characteristics of participants (preterm vs term group)^a^. **Table S2.** Comparison of preterm group vs term group^a^. **Table S3.** Laboratory findings and levothyroxine dose in preterm group^a^.

## Data Availability

The datasets used and/or analysed during the current study are available from the corresponding author on reasonable request.

## Abbreviations

CH: Congenital hypothyroidism
FT4: Free T4
NSTs: Neonatal screening tests
PCH: Permanent congenital hypothyroidism
TCH: Transient congenital hypothyroidism
TFTs: Thyroid function tests
TSH: Thyroid stimulating hormone
