# Supplementary material for: Factors associated with permanent hypothyroidism in infants with congenital hypothyroidism
Source: BMC Pediatr. 2019 Nov 22;19:453. doi: 10.1186/s12887-019-1833-8 (PMC6873549; doi:10.1186/s12887-019-1833-8)
Supplement: Supplementary file 1 — Additional file 1: Table S1. Demographic and auxologic characteristics of participants (preterm vs term group)a. Table S2. Comparison of preterm group vs term groupa. Table S3. Laboratory findings and levothyroxine dose in preterm groupa. [file 12887_2019_1833_MOESM1_ESM.docx]

**Table S1**. Demographic and auxologic characteristics of participants (preterm vs term group)^a^

| Characteristic | All patients  (n = 80) | Preterm  (n = 51) | Term  (n = 29) | *P* |
| --- | --- | --- | --- | --- |
| Male, n (%) | 40 (50.0) | 23 (45.1) | 17 (58.6) | 0.245 |
| GA (weeks) | 33.6±4.6 | 30.9±2.8 | 39.3±1.1 | <0.001^*^ |
| Age (at treatment initiation, weeks) | 3.4±3.1 | 3.8±3.5 | 2.7±2.3 | 0.131 |
| Age (at discontinuation trial, months) | 34.5±4.6 | 34.0±5.0 | 35.4±3.7 | 0.179 |
| Wt (at birth, kg) | 2.1±0.9 | 1.6±0.5 | 3.0±0.5 | <0.001^*^ |
| Wt (at treatment initiation) | 2.5±1.0 | 2.0±0.8 | 3.4±0.6 | <0.001^*^ |
| Wt (at discontinuation) | 12.8±1.8 | 12.4±1.5 | 13.6±2.1 | 0.013^*^ |

Abbreviations: Wt, weight; GA, gestational age

^a^Quantitative data are expressed as the mean ± SD (standard deviation), and qualitative data are expressed as frequency (%)

**P* < 0.05

**Table S2**. Comparison of preterm group vs term group^a^

| Characteristic | All patients  (n = 80) | Preterm  (n = 51) | Term  (n = 29) | *P* |
| --- | --- | --- | --- | --- |
|  |  |  |  |  |
| NST |  |  |  |  |
| TSH (µU/ml) (median.range) | 5.2 (0.1-356) | 4.8 (0.4-81.9) | 6.8 (0.1-356) | 0.097 |
| T4 (µg/dl) | 6.8±3.2 | 6.2±3.8 | 6.9±3.1 | 0.541 |
| TSH >20 IU/L (n,%) | 9 (13.8) | 5 (33.3) | 4 (8.0) | 0.025* |
| T4 <5 µg/dl (n,%) | 12 (21.8) | 3 (30.0) | 9 (20.0) | 0.673 |
| Initial TSH (µU/ml)(median,range) | 17.4 (0.8-100.0) | 17.3 (0.8-100.0) | 24.7 (7.2-100.0) | 0.016* |
| Initial fT4 (ng/dl) | 1.2±0.4 | 1.1±0.5 | 1.2±0.4 | 0.728 |
| TSH >20 µU/ml (n,%) | 39 (49.4) | 19 (67.9) | 20 (39.2) | 0.015* |
| fT4 <0.9 ng/dl (n,%) | 22 (30.1) | 9 (36.0) | 13 (27.1) | 0.432 |
| Delayed TSH elevation (n,%) | 20(25.0) | 16(31.4) | 4(13.8) | 0.109 |
| TSH at off trial (µU/ml) | 3.4±3.0 | 4.0±4.6 | 3.1±1.6 | 0.263 |
| fT4 at off trial (ng/dl) | 1.5±0.2 | 1.5±0.2 | 1.5±0.2 | 0.899 |
| Initial T4 dose (μg/kg/day) | 11.2±2.5 | 11.5±2.5 | 11.2±2.4 | 0.712 |
| T4 dose (1 year) (μg/kg/day) | 3.7±1.4 | 3.0±1.4 | 2.6±1.0 | 0.047* |
| T4 dose (2 years) (μg/kg/day) | 3.1±1.2 | 4.0±1.0 | 3.5±1.4 | 0.042* |
| T4 dose at off trial(μg/kg/day) | 2.8±1.2 | 3.5±1.2 | 2.9±1.1 | 0.168 |
| Off trial failure (n,%) | 9 (11.3) | 5 (17.2) | 4 (7.8) | 0.273 |

Abbreviations: Wt, weight; GA, gestational age; TSH, thyroid stimulating hormone; T4, thyroxine; fT4, free thyroxine; NST, neonatal screening test

^a^Quantitative data are expressed as the mean ± SD (standard deviation) or median (range), and qualitative data are expressed as frequency (%)

**P* < 0.05

**Table S3**. Laboratory findings and levothyroxine dose in preterm group^a^

| Characteristic | All patients  (n = 51) | Off trial failure  (n = 4) | Off trial success  (n = 47) | *P* |
| --- | --- | --- | --- | --- |
| NST |  |  |  |  |
| TSH (µU/ml)(median.range) | 4.8 (0.4-81.9) | 12.2 (0.8-61.7) | 4.8 (0.4-81.9) | 0.403 |
| T4 (µg/dl) | 6.9±3.1 | 9.0±3.3 | 6.7±3.1 | 0.238 |
| TSH >20 IU/L | 4 (8.0) | 5 (60.0) | 2(4.3) | 0.028* |
| T4 <5 µg/dl | 9 (17.6) | 0 (0.0) | 9(21.4) | 1.000 |
| Initial TSH (µU/ml)(median.range) | 17.3 (0.8-100.0) | 39.9 (1.0-74.5) | 17.3 (0.8-100.0) | 0.208 |
| Initial fT4 (ng/dl) | 1.2±0.4 | 0.8±0.1 | 1.2±0.4 | 0.085 |
| TSH >20 µU/ml | 20(39.2) | 2 (50.0) | 18 (38.3) | 1.000 |
| fT4 <0.9 ng/dl | 13(27.1) | 2 (66.7) | 11 (24.4) | 0.174 |
| TSH at off trial (µU/ml) | 3.1±31.6 | 4.4±2.6 | 2.9±1.4 | 0.348 |
| fT4 at off trial (ng/dl) | 1.5±0.2 | - | 1.5±0.2 | - |
| Initial T4 dose (μg/kg/day) | 11.2±2.4 | 10.4±3.4 | 11.3±2.3 | 0.465 |
| T4 dose (1 year) (μg/kg/day) | 3.5±1.4 | 5.2±1.4 | 3.3±1.3 | 0.011 |
| T4 dose (2 years) (μg/kg/day) | 2.9±1.1 | 3.7±1.0 | 2.8±1.1 | 0.154 |
| T4 dose at off trial (μg/kg/day) | 2.6±1.0 | 3.8±1.5 | 2.5±1.0 | 0.018* |
| Off trial failure | 4 (7.8) | 9 (100%) | 0 (0%) | - |

Abbreviations: Wt, weight; GA, gestational age; TSH, thyroid stimulating hormone; T4, thyroxine; fT4, free thyroxine; NST, neonatal screening test

^a^Quantitative data are expressed as the mean ± SD (standard deviation) or median (range), and qualitative data are expressed as frequency (%)

**P* < 0.05
